# Notes and News

**Published:** 1892-10-01

**Authors:** 


### Notes and News.

Collected and Communicated.

Derby town and county certainly distinguish themselves by moving with the times. At a recent meeting of the Town Council, it was decided to appoint a Sanitary Committee for the purpose of carrying out the provisions of the Housing of the Working Classes Act, of 1890. It is therefore to be expected that many most necessary reforms will result from this resolution.

The subject of lady students at the Cork Hospital has been taken up by a "Cork Father," who has a daughter well started in her medical career, and ultimately intending to practice amongst the women of India. Her inability to obtain instruction in the hospitals will close this career to her, her parent being unable to afford her residence where practice could be obtained. Doubtless many other fathers will be found to sympathise with a "Cork Father" in the decision of the Cork Hospital authorities.

Sir J. Maple, who some time ago presented St. Albans with an isolation hospital at a cost of £4,000, is now supplementing his generosity to that city by the munificent gift of a recreation ground and park. The principle feature on the property will be a cricket ground and pavilion, as the donor holds the results of athletics for the youth of England in high estimation. The prosperity of cricket clubs so frequently brings grist to the charity mill that we doubt not that by this second act of generosity Sir John Maple will be conferring renewed benefits on the hospitals in the neighbourhood.

A Liverpool paper of last week contained a very alarming account of the water in the Salford Docks. The chemist to the Local Sanitary Association stated that the dock water contained 42½ grains per gallon of solid matter, of which six grains were organic matter. The Manchester and Salford rivers seem to be nearly as bad; in dry weather it is stated that the water of the Irwell consists half of sewage. The Salford docks are constructed with their outlets facing downstream, and of course will have no scour. Their water will be absolutely stagnant, except from the stirring it will receive from vessels.

One more excellent institution has just been added to the roll of charity. The Heathdene Convalescent Home, at Harrogate, was opened by the Lady Mayoress of Sunderland on the 15th inst. It has been acquired for the Sunderland Infirmary, at a cost of £2,700. It is well situated, commanding pretty views all round, and is surrounded by gardens. The sitting-rooms, on the ground floor, are all 12 ft. in height, and the bedrooms 10 ft., and all are of good size. A large number of visitors attended the opening ceremony.

An appeal has just been made from the National Hospital for Paralysis, Queen's Square, for more kind friends who possess carriages to afford some of the patients, for whom fresh air is so especially beneficial, occasional carriage exercise. The idea is so reasonable that we earnestly hope it may be taken up. Many "carriage folk" will, we think, be surprised that they have never thought of such a thing before. How much pleasure might those afford, both to themselves and others, who now take their drives abroad but to exercise their horses, and frequently bore themselves in the process.

Some very sensational paragraphs are appearing giving terrible pictures of life in Metropolitan workhouses. One institution selected for opprobrium is the Lambeth old workhouse, where, it is stated, the men are given tasks which it is impossible to perform owing to defective apparatus. The other offender is the City of London Union Infirmary, Bow, where the sanitary arrangements described are truly horrible. If both accounts be true ones, reform is indeed necessary. Miss Twining has done much for the inmates of Poor Law infirmaries by introducing efficient nurses in many institutions, but much still needs to be done. The primary activity of the movement seems to have abated a little, whilst the need for proper attendance on the sick still continues. Those who have had experience of our voluntary hospitals, and, perhaps, have exercised most severe criticism, can have little conception of the discomfort that still exists in many of these State-controlled institutions.

The lady students at Cork have won the day, and they are to be admitted to the Clinical teaching at the South Infirmary. During the meeting, at which this decision was arrived at, a good deal of opposition to the movement of course arose. On the whole we are inclined to think that the remarks made by Archdeacon Archdale were more damaging to the reputation of his own sex than convincing arguments against the medical training of ladies in general hospitals. Can we suppose that the male students at the South Infirmary, who have given proofs of their chivalrous and gentlemanly feeling, will so far forget themselves as to make their presence objectionable to ladies, either by language or demeanour? Even amongst a less refined class of students we cannot but imagine that the presence of ladies would exercise a certain check, which would be by no means a disadvantage. It may be that the irksomeness of such a restraining influence has had not a little to do with the opposition to ladies entering class-rooms, where experience has revealed to us that jokes of a not very elevating nature are frequently indulged in.

